# T-Lymphocytes Activated by Dendritic Cells Loaded by Tumor-Derived Vesicles Decrease Viability of Melanoma Cells In Vitro

**DOI:** 10.3390/cimb45100493

**Published:** 2023-09-26

**Authors:** Ivan Yurevich Filin, Yuriy Pavlovich Mayasin, Chulpan Bulatovna Kharisova, Anna Valerevna Gorodilova, Daria Sergeevna Chulpanova, Kristina Viktorovna Kitaeva, Albert Anatolyevich Rizvanov, Valeria Vladimirovna Solovyeva

**Affiliations:** Institute of Fundamental Medicine and Biology, Kazan Federal University, 420008 Kazan, Russia; ivyfilin@kpfu.ru (I.Y.F.); mayasin_yuriy@mail.ru (Y.P.M.); harisovachulpan@gmail.com (C.B.K.); anagorodilova@yandex.ru (A.V.G.); daschulpanova@kpfu.ru (D.S.C.); krvkitaeva@kpfu.ru (K.V.K.); vavsoloveva@kpfu.ru (V.V.S.)

**Keywords:** dendritic cells, cytochalasin B, induced vesicles, antitumor vaccine, immunotherapy, GM-CSF, antigen presentation, cytotoxic effect

## Abstract

Immunotherapy represents an innovative approach to cancer treatment, based on activating the body’s own immune system to combat tumor cells. Among various immunotherapy strategies, dendritic cell vaccines hold a special place due to their ability to activate T-lymphocytes, key players in cellular immunity, and direct them to tumor cells. In this study, the influence of dendritic cells processed with tumor-derived vesicles on the viability of melanoma cells in vitro was investigated. Dendritic cells were loaded with tumor-derived vesicles, after which they were used to activate T-cells. The study demonstrated that such modified T-cells exhibit high activity against melanoma cells, leading to a decrease in their viability. Our analysis highlights the potential efficacy of this approach in developing immunotherapy against melanoma. These results provide new prospects for further research and the development of antitumor strategies based on the mechanisms of T-lymphocyte activation using tumor-derived vesicles.

## 1. Introduction

Cancer cells within the body possess the ability to evade the antitumor response of the immune system due to angiogenesis, fibroblasts, and suppressor cytokines that attract corresponding immune cells to the tumor area, thereby collectively forming the tumor microenvironment (TME) [1]. TME plays a fundamental role in the tumor escaping from the immune system. The presence of cells in the TME, such as suppressor cells of myeloid origin, tumor-associated macrophages, and normal regulatory T-cells suppress the activity of infiltrating cytotoxic T-lymphocytes (CTLs), which are the main effector cells of the immune system. Immunosuppressive cytokines, such as interleukin (IL)-6, IL-10, transforming growth factor (TGF)-β, and indoleamine 2,3-dioxygenase (IDO), lead to immune tolerance, increasing the number of suppressor immune cells while reducing the number of CTLs [2]. Fibroblasts, in turn, are recruited and reprogrammed by tumor cells, after which they are able to produce a variety of growth signals thereby affecting tumor growth, invasion, and metastasis [3].

Combining traditional cancer treatment methods, including surgery, chemotherapy, and radiation therapy, with immunotherapy by specifically targeting the tumor and its TME can improve treatment response rates. Immunotherapy has several advantages due to its activation of the body’s specific anti-tumor response. A therapeutic vaccine based on dendritic cells (DCs) may be a method that enhances the mechanism of a specific immune response. DCs have a crucial ability in that they cause the immune system to direct a specific immune response by internalizing foreign antigens and presenting them to CTLs. DCs are potent professional antigen-presenting cells due to the presence of the major histocompatibility complex (MHC)-II and costimulatory molecules (CD80/83/86) on their surface. Over the past two decades, DC vaccines have been shown in numerous clinical trials to be a safe therapy that can induce antitumor immunity. The most commonly observed adverse effects (AEs) include anemia, back pain, chills, fatigue, fever, headache, and nausea, generally not exceeding the 1st or 2nd grade and usually resolving within 1–2 days after vaccine administration [4]. There is currently an FDA-approved therapeutic vaccine, Sipuleucel-T, for the treatment of metastatic prostate cancer. This vaccine, based on DCs loaded with a recombinant hybrid protein (fusion of prostatic acid phosphatase (PAP) and granulocyte macrophage colony-stimulating factor (GM-CSF)), demonstrated a median improvement in overall survival of 4.1 months compared to the placebo group among men with metastatic castration-resistant prostate cancer [4]. The loading of DCs with tumor antigen is a critical component for an effective vaccine. There are several approaches to improve the efficiency of antigen presentation in order to induce a more potent immune response directed against tumor cells. The use of tumor-specific antigens (TSAs) is preferred for a more effective targeted antitumor response using unique tumor antigens. Autologous tumor mRNA, which can be delivered to DCs by electroporation [5] or viral vector [6], can be used as an antigen. However, isolating TSAs or utilizing their mRNA is energy and resource-intensive work. Moreover, TSAs for some tumors have not yet been identified. Tumor lysate is a reliable source of tumor antigens, especially for tumors whose TSAs have not been identified. In addition, the isolation of tumor lysates is a rapid and inexpensive method that can be personalized. Apart from tumor lysates, autologous tumor-derived vesicles can also serve as personalized sources of tumor antigens. Extracellular vesicles (EVs) are membrane-bound structures containing proteins and lipids as well as nuclear and mitochondrial components. EVs are able to fuse with recipient cells through endocytosis. EVs can be used to present antigens to immune system cells. However, natural EVs are not produced in sufficient amounts by tumor cells. There are alternative ways to produce EVs to overcome this problem; for example, cytochalasin B can be used. Cytochalasin B is a substance that causes disorganization of the cell cytoskeleton by preventing polymerization of its cellular structures, particularly actin filaments. Active shaking of cells treated with cytochalasin B leads to cell disintegration and formation of a large number of vesicles of different sizes. The use of cytochalasin B-induced membrane vesicles (CIMVs) may represent a novel approach of tumor antigen presentation to DCs. Furthermore, parental tumor cells from which CIMVs are derived can be modified with the cytokine gene *GM-CSF* for overexpression of the corresponding protein and subsequent immunomodulatory effects. To analyze the antigen-presenting and cytotoxic activity of immune cells, we used native CIMVs and CIMVs carrying GM-CSF derived from immortalized human melanoma M14 cells as tumor antigens to load monocyte-derived DCs (moDCs). We also loaded moDCs with CIMVs derived from triple-negative MDA-MB 231 breast cancer to compare the specific cytotoxic activity of T-cells after antigen presentation to moDCs. Also, we analyzed the cytotoxic effect of activated T-cells on human mesenchymal stem cells (MSCs).

## 2. Materials and Methods

### 2.1. Cultivation Conditions of Primary and Immortalized Human Cells

Mononuclear cells were isolated from human peripheral blood (PBMCs) of a healthy donor in a Ficoll density gradient (1.077 g/cm^3^, Cat. #P052p, PanEco, Moscow, Russia) in accordance with approved ethical standards and current legislation (protocol approved by the Biomedical Ethics Committee of Kazan Federal University (#3 dated 23 March 2017)) Informed consent was obtained from a healthy donor. The obtained blood was diluted in Dulbecco’s phosphate-buffered saline (DPBS) (Cat. #P060, PanEco, Moscow, Russia) (1:1) and then layered on Ficoll (1:1). Subsequently, centrifugation was performed at 1900 rpm for 20 min at room temperature. Mononuclear cells were collected, washed twice with PBS, resuspended in complete RPMI-1640 medium (Catalog #C330p, PanEco, Moscow, Russia) that contained 10% fetal bovine serum (FBS) (SH30071.03, HyClone, Logan, UT, USA), 2 mM L-glutamine (Catalog #F032, PanEco, Moscow, Russia), and 5000 µg/mL penicillin-streptomycin mixture (Catalog #A063p, PanEco, Moscow, Russia). Samples were then cultured in a humid atmosphere at 37 °C and 5% CO_2_. MSCs from human adipose tissue were isolated using a standard protocol [7].

The melanoma cell line M14 was obtained from the John Wayne Cancer Institute (JWCI) cell repository. Cells were cultured in a humid atmosphere at 37 °C and 5% CO_2_ in complete RPMI 1640 medium (Catalog #C330p, PanEco, Moscow, Russia). Human embryonic kidney cells HEK293T (ATCC #CRL-3216) and human triple-negative breast cancer cells MDA-MB-231 (ATCC #HTB-26) were obtained from the American Type Culture Collection (ATCC, Manassas, VI, USA). Cells were cultured in a humid atmosphere at 37 °C and 5% CO_2_ in complete DMEM with 4500 mg/l glucose (Catalog #C420p, PanEco, Russia). All cell lines were tested for the presence of mycoplasma.

### 2.2. Lentivirus Production

The donor plasmid encoding the human *GM-CSF* gene (CSF2/pENTR223) was obtained from the Harvard Plasmid Database (HsCD00365931). The *GM-CSF* gene was then recloned from the donor vector pENTR223 into the lentiviral expression vector pLX303 (AddGene #25897) using LR recombination (Catalog #11791020, Gateway™ LR Clonase™ II Enzyme mix, ThermoFisher Scientific, Waltham, MA, USA). Production of second-generation recombinant lentiviruses encoding *GM-CSF* was achieved by co-transfecting the packaging cell line HEK293T with three plasmids: the vector plasmid encoding *GM-CSF*, the packaging plasmid (psPAX2, AddGene #12260), and the envelope plasmid (pCMV-VSV-G, AddGene #8454) using calcium phosphate transfection. The culture medium was replaced with fresh medium 18 h later, followed by collection of the lentivirus-containing supernatant every 12 h for 48 h. The supernatant was stored at 4 °C during collection and then filtered through a 0.2 µm membrane (GVS filter technology, Morecambe, UK). Lentiviral particles were concentrated by ultracentrifugation for 2 h at 26,000 rpm at 4 °C.

### 2.3. Genetic Modification and Selection

The M14 cell line was transduced with the *GM-CSF* gene encoded by lentivirus in serum-free medium using 10 μg/mL protamine sulfate (#P4020, Sigma, St. Louis, MO, USA). Cells were seeded at 3 × 10^4^ cells/well in a 6-well plate and incubated with lentivirus for 6 h. Transduced cells were selected using 7.5 µg/mL blasticidin S-HCl (#R21001, Gibco, Billings, MT, USA) for 7 days. Cell culture was tested for mycoplasma and showed negative results.

### 2.4. Quantitative Polymerase Chain Reaction (qPCR)

Native and modified cell samples were resuspended in 200 µL TRIzol™ reagent (Catalog #15596026, Invitrogen, Waltham, MA, USA), followed by total RNA extraction according to the manufacturer’s recommendations. The cDNA synthesis was performed using GoScript Reverse Transcription System kit (Promega, Madison, WI, USA) according to the manufacturer’s instructions. Nucleotide sequences of primers and fluorescent probes for 18S ribosomal RNA and *GM-CSF* genes were selected using GenScript Online Real-time PCR (TaqMan) Primer Design Tool (GenScript, Piscataway, NJ, USA). Primers and probes were synthesized by Litech (Moscow, Russia) (Table 1). Real-time polymerase chain reaction (qPCR) was performed according to TaqMan technology using MicroAmp 96-well plates (Bio-Rad, Hercules, CA, USA). For the amplification reaction, 1 μL of cDNA, 0.3 μL of primer and sample mix (at a final concentration of 300 nM), 4.7 μL of MilliQ H_2_O, and 4 μL of 10x TaqMan buffer (Eurogen, Russia) were used. The final reaction volume was 10 μL. Amplification was performed on a CFX96 Touch™ Real-Time PCR Detection System (Bio-Rad, USA) using the following temperature profile: denaturation at 95 °C for 1 min; 44 cycles including denaturation at 95 °C for 30 s; primer annealing at 55 °C for 30 s; and elongation at 72 °C for 1 min.

### 2.5. Western Blot Analysis

Samples of native and modified cells (1 × 10^6^ cells) were lysed using RIPA buffer (#89900, ThermoFisher Scientific, USA) containing a protease and phosphatase inhibitor mixture (#78444, ThermoFisher Scientific, USA). Protein concentration was determined using the Pierce™ BCA Protein Assay Kit (#23225, ThermoFisher Scientific, USA). Equal amounts of protein were denatured on a dry bath at 95 °C for 5 min, loaded and separated on 12% SDS-PAGE gels, and then transferred to PVDF membranes. The membranes were preblocked in 5% BCA solution (ThermoFisher Scientific, USA) and then incubated with primary antibodies at 4 °C overnight, followed by washing with Phosphate Buffered Saline with Tween™ 20 (#P1379, Sigma, USA) (PBS-T) and incubation with secondary antibodies at room temperature for 2 h. The membranes were then washed with PBS-T, and the target protein band was visualized using HRP (BioRad, Hercules, CA, USA) and analyzed using the ChemiDoc XRS+ system (BioRad Laboratories, Irvine, CA, USA). Primary rabbit polyclonal antibodies to GM-CSF (1:500) were purchased from Abcam, Cambridge, UK (ab300495). Secondary goat anti-rabbit IgG H&L (Alkaline Phosphatase) antibodies (1:2000) were purchased from Abcam, UK (ab97048).

### 2.6. Immunocytochemistry

Immunocytochemical analysis was performed to analyze GM-CSF protein synthesis in native M14 and M14-GM-CSF cells. Cells were seeded in 24-well plates (1 × 10^3^ cells/well) on coverslips in complete RPMI-1640 medium. On the next day, the culture medium was aspirated, and cells were fixed with chilled methanol for 10 min at room temperature. After fixation, cells were washed with Tris-buffered saline (TBS) three times for 5 min each. The cells were then incubated with primary antibodies against GM-CSF (Cat. #ab54429, Abcam, Cambridge, UK, dilution 1:100 in TBS) for 1 h at room temperature. The stained cells were washed three times for 5 min each with TBS to remove unbound antibodies, and then incubated with secondary antibodies (Goat anti-Rabbit IgG (P&L) Fluorescein, Catalog #A102FN, American Qualex, San Clemente, CA, USA; dilution 1:1000 in TBS) for 1 h at room temperature, followed by washing. DAPI fluorescent dye (4′,6-diamidino-2-phenylindole; 1:50,000 dilution in TBS; Cat. #D1306, Invitrogen, USA) was used to stain the nucleus, then incubated for 5 min at room temperature, followed by washing. The coverslips were mounted on slides using mounting medium (ImmunoHistoMount, Catalog #sc-45086, Santa Cruz Biotechnology, Dallas, TX, USA). The analysis was conducted using a confocal microscope LSM 780 (Carl Zeiss, Jena, Germany) and ZEN (black edition) 2.3 software (Carl Zeiss, Germany).

### 2.7. Isolation of Cytochalasin-Induced Membrane Vesicles (CIMVs)

Cells were washed from FBS with PBS (Catalog #P060, PanEco, Russia). After counting, 2 × 10^6^ cells were suspended in 10 mL serum-free medium containing cytochalasin B from Drechslera dematioidea (#C6762-5mg, Sigma-Aldrich, St. Louis, MO, USA) at a concentration of 10 µg/mL. Cells were incubated in a humid atmosphere for 30 min at 37 °C and 5% CO_2_ with gentle shaking every 10 min. After incubation, cells were vortexed for 1 min, then centrifuged for 10 min at 700 rpm (Biosan LMC-3000 laboratory centrifuge). The supernatant was collected and centrifuged for 10 min at 1400 rpm; then, the supernatant was collected and filtered through a polyvinylidene difluoride (PVDF) membrane filter (GVS filter technology, UK) with a pore size of 1 micron. After filtration, the supernatant was centrifuged for 30 min at 12,000 rpm. The resulting pellet containing CIMVs was collected. Protein concentration was determined using the Pierce BCA Protein Assay Kit (#23225, ThermoFisher Scientific, USA) according to the manufacturer’s instructions.

### 2.8. Size Characterization of Cytochalasin-Induced Membrane Vesicles (CIMVs)

To determine the size of CIMVs, scanning electron microscopy was used. Isolated CIMVs, as described earlier, were deposited onto glass slides in the wells of a 96-well plate by centrifugation at 3000 rpm for 30 min at room temperature. The deposited CIMVs were fixed in 10% formalin for 15 min, dehydrated with ethanol increasing in concentration from 30% to absolute, and then air-dried for 24 h. Before visualization, the samples were coated with palladium using the Quorum T150ES sputter coater (Quorum Technologies Ltd., Lewes, UK), and analysis was performed using the SEM Merlin (Carl Zeiss, Germany). Additionally, flow cytometry (BD FACSAria III, BD Biosciences, San Jose, CA, USA) was used to measure the size of CIMVs using a mixture of calibration particles (0.22–0.45–0.88–1.34 µm) (Spherotech, Lake Forest, IL, USA). At least 50,000 events were acquired for each sample.

### 2.9. Assessment of Nuclear, Membrane, and Mitochondrial Components in CIMVs

To analyze the presence of nuclear, membrane, and mitochondrial components in CIMVs, native M14, M14-GM-CSF, and MDA-MB 231 cells were trypsinized and washed in PBS twice. Next, 5 × 10^5^ cells were suspended in 1 mL of PBS and stained using 1 μL of DiO vital dye (Vybrant Multicolor Cell-Labeling Kit, Invitrogen, Waltham, MA, USA) for 10 min at room temperature and in the absence of light. Subsequently, the cells were washed twice and resuspended in 500 μL of PBS, followed by staining with 2 μL of 300 nM MitoTracker Red FM (Molecular probes, Invitrogen, Waltham, MA, USA) for 15 min at 37 °C. Afterward, the cells were again washed twice with PBS, and CIMVs were isolated as described earlier. These isolated CIMVs were fixed in cold methanol for 5 min at room temperature, then resuspended in 500 μL of PBS and stained using 0.5 μL of 7-aminoactinomycin D (7AAD) (Molecular probes, Invitrogen, Waltham, MA, USA) for 10 min at room temperature in the absence of light. The isolated CIMVs were analyzed using FACS Aria III (BD Biosciences, USA) and BD FACSDiva^TM^ software version 7.0.

### 2.10. Assessment of Surface and Intracellular Markers in CIMVs

To analyze the expression of the cluster of differentiation (CD) markers typical for tumor cells on the surface of the CIMVs, the following antibodies were used: PE/Cyanine7 anti-human CD81 (#349512, Biolegend, San Diego, CA, USA), Alexa Fluor^®^ 488 anti-human Hsp70 (#648004, Biolegend, USA), PerCP/Cyanine5.5 anti-human CD63 (#353020, Biolegend, USA), PE anti-human TSG101 (ab209927, abcam, USA), Alexa Fluor^®^ 594 anti-human Calnexin (ab203439, abcam, USA). Briefly, CIMVs were isolated from 2 × 10^5^ native M14, M14-GM-CSF, and MDA-MB 231 cells that were pre-fixed in cold methanol for 5 min at room temperature, washed once in PBS and stained with antibodies for 30 min at room temperature in the absence of light. Then, CIMVs were washed with PBS and analyzed using the FACSAria III flow cytometer (BD Biosciences, San Jose, CA, USA) and BD FACSDiva™ software version 7.0.

### 2.11. Generation of Dendritic Cells from Monocytes

Monocytes were isolated from healthy donor PBMCs by adhesion. They were seeded at a density of 10 million cells per well and incubated in a humid atmosphere at 37 °C and 5% CO_2_ for 1.5 h. After this, non-adherent cells were removed along with the medium, and fresh complete RPMI-1640 medium (Catalog #C330p, PanEco, Russia) which contained 10% FBS, 2 mM L-glutamine and 1% autologous plasma was added. The generation of moDCs was carried out in 6-well plates. On day 0, isolated monocytes (5 × 10⁶ cells/well) were cultured in 3 mL of complete RPMI-1640 medium supplemented with 250 IU/mL IL-4 (Catalog #PSG040-10, SCI-Store, Moscow, Russia), and 800 IU/mL GM-CSF (Catalog #PSG030-10, SCI-Store, Russia) at 37 °C and 5% CO_2_. On days 2 and 5, 1.5 mL of the medium was collected, centrifuged, and the cell pellet was resuspended in 1.5 mL of complete medium supplemented with double concentration of IL-4 and GM-CSF. This mixture was returned to the original culture. On day 8, 1.5 mL of the medium was collected, centrifuged, and the cell pellet was resuspended in 1.5 mL of medium supplemented with 2000 IU/mL IL-6 (Catalog #PSG180-10, SCI-Store, Russia), 400 IU/mL IL-1β (RPA563Hu01, Cloud-Clone Corp., Katy, Houston, TX, USA), 2000 IU/mL TNF-α (Catalog #PSG250-10, SCI-Store, Russia), and 2 μg/mL prostaglandin E2 (PGE2) (#900117P-5MG, Sigma-Aldrich, USA). Additionally, 15 μg/mL of CIMVs were added to the culture. The cells were then cultured for an additional 48 h. On day 9, the viability, yield, and absolute cell count of mature moDCs were determined by flow cytometry using specific surface markers (see Section 2.10). Subsequently, mature moDCs were re-seeded in 6-well plates for antigen presentation with fresh PBMCs.

### 2.12. Immunophenotyping of Monocyte-Derived Dendritic Cells after Differentiation from Monocytes

Sensitive conjugated antibodies were used to determine the target population of mature moDCs. Cells were removed from the culture plate and washed from the medium with PBS. The moDCs were then stained with conjugated antibodies: Pacific Blue™ anti-human CD3 (#300330, Biolegend, USA), Pacific Blue™ anti-human CD19 (#302232, Biolegend, USA), Pacific Blue™ anti-human CD56 (#362520, Biolegend, USA), Pacific Blue™ anti-human CD20 (#302328, Biolegend, USA), PerCP/Cyanine5.5 anti-human CD11c (#301624, Biolegend, USA), FITC antibody to human HLA-DR (#307604, Biolegend, USA), APC anti-human CD64 (#305014, Biolegend, USA), PE anti-human CD80 (#305208, Biolegend, USA), PE anti-human CD83 (#305308, Biolegend, USA) according to the manufacturer’s instructions. Data were analyzed using FACS Aria III (BD Biosciences, USA) and BD FACSDiva^TM^ software version 7.0.

### 2.13. Analysis of the Interaction between Cytochalasin B-Induced Membrane Vesicles and Monocyte-Derived Dendritic Cells

To avoid loss of CIMVs during staining, M14 cells were pre-stained with CellTracker™ Green CMFDA dye (#C7025, Invitrogen, USA) according to the manufacturer’s instructions. After staining, CIMVs were isolated according to the previously described protocol. The moDCs were stained with the Vybrant™ Multicolor Cell-Labeling Kit (#V-22889, Thermo Fisher Scientific, USA) using DiD spectrum according to the manufacturer’s instructions and seeded in a 12-well culture plate. The moDCs were incubated with CIMVs for 3 h in a humidified atmosphere at 37 °C and 5% CO_2_. The moDCs were settled by centrifugation at 1400 rpm for 5 min. The cells were then washed from the growth culture medium with PBS and deposited at the bottom of the wells of the culture plate onto a coverslip. At this point, it is important to ensure that the coverslip remains at the bottom of the well. This procedure was followed by incubation for 30 min at room temperature for deposition and attachment of moDCs to the coverslips. After cell attachment, PBS was carefully removed, and cells were fixed with 500 μL of 10% formalin for 10 min at room temperature. The fixed cells were first washed once with 500 μL of PBS for 5 min and then permeabilized with 500 μL of 0.5% Triton X-100 for 10 min. The permeabilized cells were washed once more with 500 μL PBS for 5 min. The nuclei were stained with DAPI (dilution 1:10,000) for 10 min, then washed twice with PBS for 5 min to remove excess dye. For sample preparation, coverslips were carefully mounted on microscope slides using aqueous mounting medium (ab128982, Abcam, UK). Samples were analyzed by confocal microscopy using a LSM 780 confocal microscope and ZEN (black edition) 2.3 software (Carl Zeiss, Germany) at the KFU Interdisciplinary Center for Analytical Microscopy.

### 2.14. Analysis of Surface Markers of Peripheral Blood Mononuclear Cells after Culturing with Activated Mature Monocyte-Derived Dendritic Cells

The isolated PBMCs were seeded in a 6-well culture plate with activated mature moDCs at a ratio of 1:30. Native PBMCs were used as controls. Flow cytometry analysis was performed after 72 h of incubation. Also, some of these PBMCs were used for tumor cell apoptosis analysis after 72 h of co-culture, respectively. Conjugated antibody staining was performed to detect activated immune cell populations. Cells were divided into panels and stained with sensitive conjugated antibodies: FITC anti-human CD8a (#300906, Biolegend, USA), APC anti-human CD4 (#357408, Biolegend, USA), PE/Cy7 anti-human CD38 (#356608, Biolegend, USA), PE anti-human HLA-DR (#307605, Biolegend, USA), Brilliant Violet 421™ anti-human CD107a (LAMP-1) (#328626, Biolegend, USA), FITC anti-human CD3 (#300306, Biolegend, USA), Pacific Blue™ anti-human CD4 (#317429, Biolegend, USA), PE anti-human CD127 (IL-7Rα) (#351304, Biolegend, USA), PE/Cyanine7 anti-human CD25 (#356108, Biolegend, USA), PE anti-human CD196/CCR6 (#353410, Biolegend, USA), APC anti-human CD183/CXCR3 (#353708, Biolegend, USA), PerCP/Cyanine5.5 anti-human CD56/NCAM (#362506, Biolegend, USA) according to the manufacturer’s instructions. Data were analyzed using FACS Aria III (BD Biosciences, USA) and BD FACSDiva^TM^ software version 7.0.

### 2.15. Assessment of Tumor Cell Viability

Native M14 melanoma cells were seeded in a 12-well culture plate (5 × 10^4^ cells per well) preliminarily 24 h before the apoptosis assay. MSCs cells were also seeded in the same amount to test for nonspecific cytotoxicity. Then, activated PBMCs (2.5 × 10^5^ cells per well) were added to M14 cells and MSCs. M14 and MSC cells were harvested and washed with DPBS after 24 h. APC Annexin V Apoptosis Detection Kit with PI (#640932, Biolegend, USA) was used for apoptosis assay according to the manufacturer’s instructions. Data were analyzed using FACS Aria III (BD Biosciences, USA) and BD FACSDiva^TM^ software version 7.0.

### 2.16. Statistical Analysis

GraphPad Prism 8 software (GraphPad Software, San Diego, CA, USA) was used for statistical analysis. One way ANOVA analysis was used to compare independent groups by quantitative characteristics. Differences between groups were considered statistically significant at *p* < 0.05, *p* < 0.0001.

## 3. Results

### 3.1. Production of Genetically Modified Melanoma Cells Overexpressing GM-CSF

**Figure 1:**
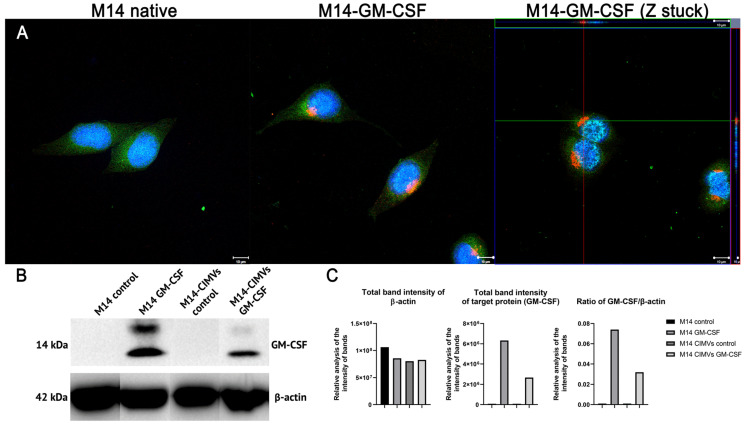
Characterization of native and genetically modified M14 melanoma cells. (**A**) Immunofluorescence analysis of GM-CSF synthesis in M14 (blue DAPI, green DIO, red monoclonal antibody (mAb) to GM-CSF) (Scale bar: 10 μm); (**B**) Western blot analysis of GM-CSF expression in M14 melanoma cells and CIMVs using β-actin as an internal control; (**C**) The relative analysis of the intensity of protein bands.

Genetically modified human melanoma M14 cells were obtained by lentiviral transduction. Confirmation of GM-CSF expression was demonstrated by qPCR and Western blot. Elevated levels of GM-CSF gene transcripts (78.3 gene copies per cell, standard deviation (SD) ± 10.12) were demonstrated in M14-GM-CSF compared to the absence of such in native M14 cells. Membrane vesicles were isolated from both native and genetically modified M14 cells using cytochalasin B treatment. The presence of GM-CSF protein in cells and CIMVs was confirmed by Western blot analysis (Figure 1B). The data obtained indicate that modified M14 tumor cells and CIMVs isolated from them contain GM-CSF protein with a molecular mass of 14 kDa. This protein was absent in native M14 tumor cells and isolated CIMVs. The presence of GM-CSF protein in M14 cells was also confirmed by immunofluorescence analysis (Figure 1A).

### 3.2. Preparation and Analysis of Induced Membrane Vesicles from Native and Genetically Modified Tumor Cells

**Figure 2:**
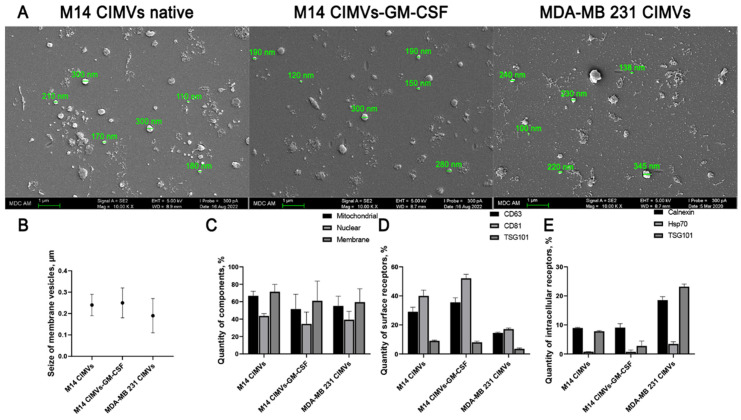
Analysis of the presence of intracellular components and the size of cytochalasin B-induced membrane vesicles isolated from native M14, M14-GM-CSF and MDA-MB 231. The size of induced membrane vesicles was determined by (**A**) scanning electron microscopy (scale bar: 1 μm) and (**B**) flow cytometry; (**C**) The presence of mitochondrial, nuclear and cytoplasmic components and the presence of (**D**) cell surface and (**E**) intracellular receptors in CIMVs were analyzed by flow cytometry.

Induced membrane vesicles were isolated from native and genetically modified M14 tumor cells as well as from MDA-MB 231 cells by treating the cells with cytochalasin B. It was shown that the size of isolated CIMVs from M14 averaged 240 nm (SD ± 70), from M14-GM-CSF averaged 250 nm (SD ± 50), and from MDA-MB 231 averaged 190 nm (SD ± 80) (Figure 2B).

Scanning electron microscopy (SEM) was used to examine the shape and size of CIMVs (Figure 2A). They had a characteristic spherical shape and varied in size. It is important to note that some deformation of vesicles was observed, which is a common phenomenon when preparing samples for SEM analysis and is not an artifact of the vesicle isolation process using cytochalasin B.

Furthermore, flow cytometry data indicated the presence of nuclear components in the following averages: M14 CIMVs—43.6% (SD ± 2.75), M14 CIMVs-GM-CSF—34.45% (SD ± 13.7), MDA-MB 231 CIMVs—39.4% (SD ± 9.5). Additionally, mitochondrial components were observed with the following averages: M14 CIMVs—66.8% (SD ± 5.15), M14 CIMVs-GM-CSF—51.5% (SD ± 17.1), MDA-MB 231 CIMVs—55.15% (SD ± 11.2). Cytoplasmic components were also present, with the following averages: M14 CIMVs—71.4% (SD ± 8.4), M14 CIMVs-GM-CSF—61% (SD ± 22.7), MDA-MB 231 CIMVs—59.5% (SD ± 15.22) (Figure 2C).

Upon staining vesicles for typical tumor biomarkers, the presence of surface receptors CD63, CD81, and TSG-101 was demonstrated. The averages for M14 CIMVs were 29% (SD ± 3), 40% (SD ± 3.95), and 9% (SD ± 0.46). The averages for M14 CIMVs-GM-CSF were 35.5% (SD ± 3), 52% (SD ± 2.76), and 8% (SD ± 0.78). And the averages for MDA-MB 231 CIMVs were 14.5% (SD ± 0.45), 17% (SD ± 0.7), and 3.5% (SD ± 0.6), respectively (Figure 2D). Additionally, the presence of intracellular receptors Calnexin, Hsp70, and TSG-101 was demonstrated. The averages for M14 CIMVs were 8.9% (SD ± 0.25), 0.77% (SD ± 0.06), and 7.8% (SD ± 0.3). The averages for M14 CIMVs-GM-CSF were 9.1% (SD ± 1.35), 0.73% (SD ± 0.67), and 2.77% (SD ± 1.7). And the averages for MDA-MB 231 CIMVs were 18.6% (SD ± 1.16), 3.47% (SD ± 0.75), and 23.2% (SD ± 0.92), respectively (Figure 2E).

### 3.3. Generation and Characterization of Dendritic Cells from the Population of Human Peripheral Blood Monocytes

**Figure 3:**
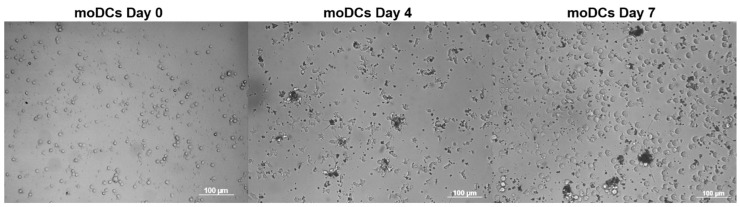
Mature monocyte-derived dendritic cells obtained after monocyte differentiation on the seventh day of cultivation (bright-field microscopy). Scale bar: 100 µm.

As a result of targeted differentiation, rounded and dendritic morphology was observed in the moDCs generated (Figure 3).

**Figure 4:**
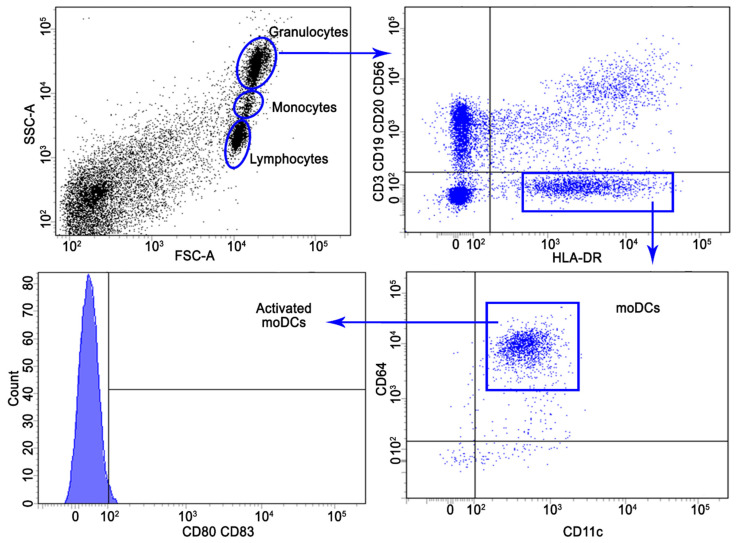
Gating strategy for the identification of CD3^−^CD19^−^CD20^−^CD56^−^HLA-DR^+^CD64^+^CD11c^+^ moDCs and activated moDCs (CD80^+^CD83^+^) population of PBMCs.

An immunophenotypic analysis was performed using flow cytometry with conjugated antibodies to confirm the phenotype of mature dendritic cells. As an example, the strategy for gating a population of moDCs from PBMCs used during flow cytometry is shown in Figure 4.

**Figure 5:**
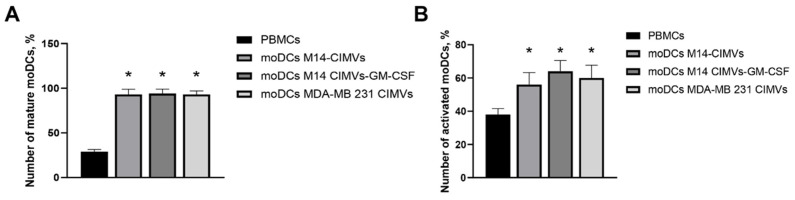
(**A**) Evaluation of differentiation efficiency of mature moDCs loaded with M14 CIMVs, M14 CIMVs-GM-CSF and MDA-MB 231 CIMVs; (**B**) Number of activated moDCs among the DC population. *—compared to the control group, * *p* < 0.0001.

During the analysis of surface markers on mature moDCs, an increase of 65% in the population of moDCs differentiated from the monocyte population compared to control PBMCs was observed. Moreover, activated moDCs expressing CD80 and CD83 co-stimulatory domains loaded with M14 CIMVs, M14 CIMVs-GM-CSF and MDA-MB 231 CIMVs among differentiated moDCs were 56% (SD ± 7.24), 64% (SD ± 6.55) and 60% (SD ± 7.67), respectively (Figure 5A), while the number of activated moDCs among the population of DC in PBMCs culture was 38% (SD ± 3.59) (Figure 5A).

### 3.4. Analysis of the Interaction between Induced Membrane Vesicles and Monocyte-Derived Dendritic Cells

**Figure 6:**
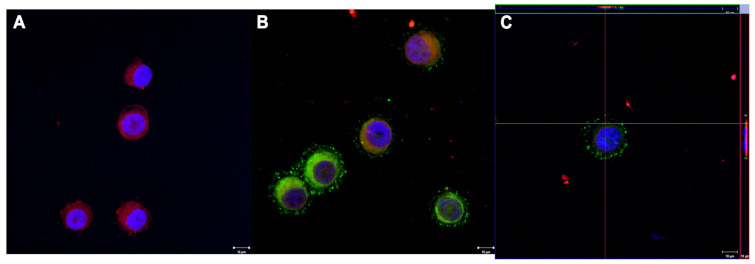
Confocal Microscopy (Scale 10 μm). (**A**) Control moDCs (Red DID, Blue DAPI); (**B**) moDCs (Red DID, Blue DAPI) after cultivation with CIMVs (Green DIO); (**C**) Z-stack moDCs (Red DID, Blue DAPI) after cultivation with CIMVs (Green DIO).

To analyze the interaction of moDCs with tumor-derived CIMVs, moDCs prestained with vital dyes were added to the bottom of the wells of the culture plate for 3 h. The moDCs were then washed to remove any vesicles, seeded on coverslips, and prepared for confocal microscopy. As a result, a clear interaction between moDCs and CIMVs was demonstrated, characterized by the uptake of CIMVs by monocyte-derived dendritic cells through endocytosis or fusion processes (Figure 6).

### 3.5. Analysis of Mononuclear Cell Activation and Cytotoxic Efficacy after Co-Culture with Activated Monocyte-Derived Dendritic Cells

**Figure 7:**
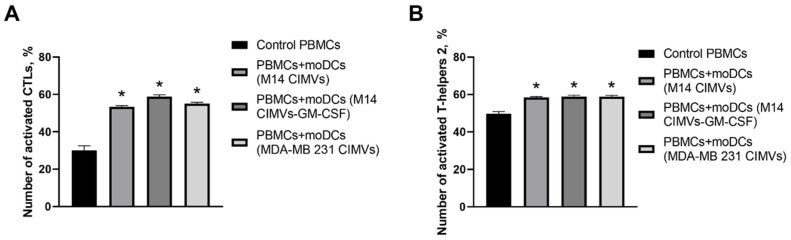
Comparative assessment of CTLs (**A**) and Th2 (**B**) activation by dendritic cells loaded with M14 CIMVs, M14 CIMVs-GM-CSF, and MDA-MB 231 CIMVs. *—compared to the control group, *p* < 0.0001.

An increase in the number of HLA-DR^+^/CD38^+^ cytotoxic T-lymphocytes (CTLs) was observed in all experimental samples (Figure 7A). However, co-culture of moDCs loaded with M14 CIMVs-GM-CSF with PBMCs led to a 29% increase (95% confidence interval (CI) of 25.03 to 32.57) in HLA-DR^+^/CD38^+^ CTLs compared to control PBMCs. Additionally, an increase in the number of T-helper 2 (Th2) cells was demonstrated in all experimental samples, showing a 10% increase (95% CI 6.87 to 11.39) compared to control cells (Figure 7B).

**Figure 8:**
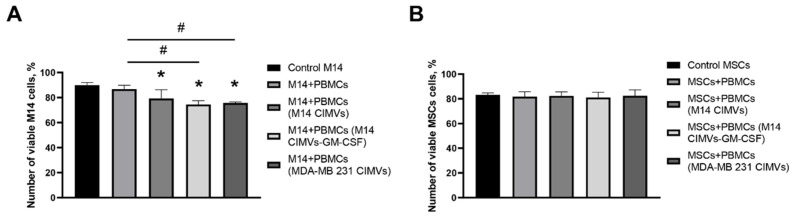
The number of viable M14 cells (**A**) and MSCs (**B**) after 24 h of cultivation with non-activated PBMCs or PBMCs activated by moDCs loaded with M14-CIMVs, M14-CIMVs-GM-CSF, and MDA-MB 231-CIMVs. *—compared to the control group, *p* < 0.0001; #—*p* < 0.0001.

To determine cytotoxic effectiveness towards M14 cells, unactivated PBMCs and PBMCs activated by DCs loaded with M14 CIMVs, M14 CIMVs-GM-CSF, and MDA-MB 231 CIMVs were added. Similarly, to assess nonspecific cytotoxicity, the same PBMCs were added to MSCs. PBMCs with tumor cells and MSCs were cultured for 24 h. Untreated M14 cells and MSCs were used as controls. It was observed that the presence of activated PBMCs (M14 CIMVs) and PBMCs (M14 CIMVs-GM-CSF) led to a reduction in the number of viable M14 cells by 10.5% (95% CI of 5.18 to 15.71) and 15.3% (95% CI of 10.07 to 20.60), respectively, compared to control cells. Furthermore, when PBMCs (MDA-MB 231 CIMVs) were added to M14 cells, a decrease in viable cell count was also observed by 14% (95% CI of 7.52 to 20.65) compared to control M14 cells and by 11% (95% CI of 4.74 to 17.33) compared to coculture of M14 cells and unactivated PBMCs (Figure 8A). When unactivated and activated PBMCs were added to MSCs, no statistically significant differences were found compared to control MSCs (Figure 8B).

## 4. Discussion

Under natural conditions, extracellular vesicles (EVs) serve as crucial transport mediators between cells, facilitating the transfer of various proteins, lipids, and nucleic acids. They can also regulate gene expression in recipient cells, as well as have internal effects on regulation of gene expression in parental cells [8]. Functioning as mediators of intercellular communication by carrying molecules from parent cells, EVs are considered promising tools for drug delivery and presentation of foreign antigens in cancer treatment [9]. However, obtaining a sufficient quantity of natural EVs for necessary research is a laborious task as these EVs are released by cells in limited amounts. In order to facilitate the isolation procedure and increase the yield of EVs, we treated tumor cells with cytochalasin B in our study.

Most commonly, the sizes of the membranous vesicles derived from tumor cells, including exosomes, range from 30 to 150 nm, while the size of the microvesicles ranges from 100 to 1000 nm [10,11,12]. In our study, the average size of isolated CIMVs from M14 cells was 240 nm, while CIMVs from M14-GM-CSF cells measured around 250 nm. The average size of the isolated CIMVs from MDA-MB 231 cells was approximately 190 nm. The CIMVs we investigated showed positive expression of typical EV biomarkers such as CD63, CD81, HSP70, TSG-101, and Calnexin. It is notable that MDA-MB 231 CIMVs had more intracellular markers than surface markers, while for M14 CIMVs, the pattern was reversed. The presence of these markers indicates that the studied CIMVs are tumorigenic [11,13]. Nuclear, mitochondrial, and lipid components were also analyzed. It’s noted that tumor-derived exosomes contain a variety of genetic elements, such as DNA, mRNA, miRNA, proteins, and lipids [10]. The presence of mitochondrial components in CIMVs was, on average, from 51–66%, nuclear component was found in 34–44% of cases, and the membrane component of vesicles was from 59–71% of cases.

Dendritic cells were derived from human peripheral blood monocytes using growth factors such as GM-CSF and IL-4. A cytokine cocktail including PGE2, IL-1β, IL-6, and TNF-α was used to mature moDCs. This cytokine cocktail combining several protein factors leads to an improved immune response [2]. Immunophenotypic analysis showed high levels of moDC-specific receptor expression. These cells had a round dendritic morphology with reduced adhesion (Figure 3), which is consistent with data from the literature [14].

The protein GM-CSF was chosen as an immunomodulatory agent due to its ability to provide additional stimulation to moDCs. The moDCs activated in this way promote the differentiation of naive CD4^+^ T-cells into T-helper subsets and also contribute to the development of CD8^+^ T-cell responses [15,16,17,18]. Furthermore, this cytokine can serve as a potential immune adjuvant in inducing an antitumor response [18]. Thus, it was decided to use tumor cells overexpressing GM-CSF to isolate CIMVs. The presence of the protein in cells and vesicles was confirmed by immunofluorescence and Western blot analyses (Figure 1). In addition, CIMVs uptake by moDCs via endocytosis or fusion was shown by immunofluorescence analysis, which is correlated with literature data [19]. In our study, we found that a higher quantity of co-stimulatory molecules was present on the surface of moDCs loaded with CIMVs-GM-CSF, as well as an increase in the number of activated CTLs after antigen presentation by these moDCs. However, no statistically significant differences were observed among the experimental groups.

Cytotoxic CD8^+^ T-cells play a critical role in anti-tumor immune responses [20]. Due to the ability of DCs to present tumor antigen to CD8^+^ T-cells, tumor-derived CIMVs may be potential carriers of tumor-specific antigens. Tumor-derived exosomes from human melanoma cells promote the maturation of bone marrow derived DCs, resulting in the induction of proliferation of dendritic cell-processed T-cells [21]. By analyzing specific surface markers for CTLs and T-helpers, we observed that the culture of CD8^+^ T-cells with moDCs loaded with M14 CIMVs, CIMVs-GM-CSF, or MDA-MB 231 CIMVs leads to a significant increase in activated CD38^+^ HLA-DR^+^ killer T-cells compared to the control group. Notably, a more significant increase in the number of CD38^+^ HLA-DR^+^ T-killers was in the group with moDCs loaded with M14 CIMVs-GM-CSF (2-fold). At the same time, an increase in Th2 number was observed in all experimental groups compared to the control (1.2 times). The transcriptional profile of DCs maturing under the influence of TNF-α/CD40L demonstrates that such DCs polarize T-cells towards a Th2 response [2]. It is known that a high expression of Th1 gene is associated with a favorable and non-recurrent outcome [22,23,24,25]. Meanwhile, a Th2 response has been linked to tumor immune evasion in mouse studies [26,27]. However, there are studies showing that the main role of Th2 cells in patient prognosis has not been confirmed [28]. It is known that the provision of an antitumor response by Th2 cells strongly depends on the type and stage of an individual tumor [29]. In particular, adoptive therapy using Th2 cells has already been shown to be effective in animal models of melanoma [30]. Moreover, there are studies showing that Th2 cells play an important role in inhibiting the progression of colon and pancreatic cancer in mice by recruiting eosinophils to tumors where they produce cytotoxic factors [31]. Thus, using the cytotoxic activity of eosinophils Th2 cells has great potential in the antitumor response [32].

In order to evaluate the efficiency of CD8^+^ and CD4^+^ T-cell activation, we performed cytotoxic activity assay. After 24 h of culturing activated PBMCs with M14 melanoma cells, we observed a decrease in the number of viable cells in all experimental samples compared to untreated M14 cells, except for the co-culture of M14 cells with unactivated PBMCs. Similar data indicate that human DCs loaded with apoptotic tumor cells and subsequently matured efficiently generated T cell-mediated antitumor responses in vitro [33]. Interestingly, PBMCs activated by moDCs-MDA-MB 231-CIMVs exhibited similar cytotoxic activity against M14 melanoma cells as PBMCs activated by moDCs-M14-CIMVs and moDCs-M14-CIMVs-GM-CSF. These findings suggest that CIMVs obtained from breast cancer and melanoma cells likely carry certain tumor-associated antigens (TAAs). It is also known that tumor-specific CTL clones can recognize breast cancer and melanoma cells [34]. Moreover, various members of the melanoma antigen (MAGE)-A and -C gene subfamilies are typically aberrantly activated in breast cancer [35]. However, co-culture of activated PBMCs with MSCs for 24 h showed no significant difference compared to untreated MSCs, indicating the absence of a nonspecific cytotoxic response. Thus, these results suggest the ability of moDCs loaded with tumor-derived CIMVs to activate CD8^+^ T-killers, and the generated T-killers are able to kill human melanoma tumor cells in vitro.

## 5. Conclusions

In conclusion, this study demonstrates the potential of dendritic cell-based therapies using tumor-specific CIMVs to activate CD8^+^ cytotoxic T-lymphocytes and initiate effective anti-tumor responses. These results reveal new evidence of complex interactions between immune cell populations and tumors, providing new potential opportunities to develop novel and targeted immunotherapeutic approaches to more effectively combat cancer. Furthermore, research is needed to fully explore the potential of these immune responses and develop personalized and efficient methods for cancer treatment.

## Figures and Tables

**Table 1:** Nucleotide Sequences of Primers and Fluorescent Probes.

Gene	Forward Primer (5′–3′)	Reverse Primer (5′–3′)
*18S*	GCCGCTAGAGGTGAAATTCTTG	CATTCTTGGCAAATGCTTTCG
*GM-CSF*	GCGTCTCCTGAACCTGAGTA	CCCTGCTTGTACAGCTCCAG

## Data Availability

Authors can confirm that all relevant data used to support the findings of this study are including within the article.
